# Short-Term Cardiac and Noncardiac Mortality Following Liver Transplantation

**DOI:** 10.1155/2010/910165

**Published:** 2010-08-12

**Authors:** Mackram F. Eleid, R. Todd Hurst, Hugo E. Vargas, Jorge Rakela, David C. Mulligan, Christopher P. Appleton

**Affiliations:** ^1^Department of Internal Medicine, Mayo Clinic, Scottsdale, AZ 85259, USA; ^2^Division of Cardiovascular Diseases, Department of Internal Medicine, Mayo Clinic, Scottsdale, AZ 85259, USA; ^3^Division of Hepatology, Department of Internal Medicine, Mayo Clinic, Scottsdale, AZ 85259, USA; ^4^Division of Transplant Surgery, Department of Internal Medicine, Mayo Clinic, Scottsdale, AZ 85259, USA

## Abstract

*Objectives*. To determine the importance of acute cardiac events as a cause of mortality compared to non-cardiac events in the four month period following liver transplantation (LT) using current preoperative cardiac screening strategies. *Patients and Methods*. We retrospectively reviewed timing, type, and outcome of adverse cardiac events, and all cause mortality in the 4 month postoperative period in 393 consecutive LT patients from October 1999 to February 2008. *Results*. Of 30 total deaths (7.6% overall mortality rate), 27 (90%) were due to surgical or medical complications and 3 (10%) were primary cardiac deaths (0.8% cardiac mortality rate). Acute cardiac events occurred in 26 patients (6.6%), including 13 arrhythmias (50%), 7 new onset heart failures (27%), and 6 myocardial infarctions (23%). Twelve of 13 intraoperative events were arrhythmias (92%) including two of three cardiac deaths. *Conclusions*. Using current preoperative screening recommendations, deaths from primary cardiac events within four months of LT are very uncommon (0.8%), especially compared with deaths related to medical and surgical complications (6.9%).

## 1. Introduction

Acute cardiac events such as myocardial infarction, congestive heart failure, and arrhythmias are an important cause of death in end stage liver disease (ESLD) patients both at the time of and after liver transplantation (LT). However, identifying LT candidates at risk for adverse cardiac events remains a challenge. Traditional risk stratification algorithms such as the Framingham Risk Score (FRS) are poor predictors of cardiac events in the ESLD population [1]. The American Association for the Study of Liver Diseases (AASLD) recommends that LT candidates who are older than 50, diabetic, past or present smokers, or have a family history of heart disease undergo a cardiac stress test (dobutamine stress echo (DSE), pharmacologic nuclear perfusion) or cardiac catheterization to rule out obstructive coronary artery disease (CAD) [2]. However, the data to support this recommendation is controversial [3–6] and whether this strategy is effective in reducing cardiac events following LT has not been proven. In addition current evidence [7–9], including our own experience [10], suggests that serious postcardiac arrhythmias at the time of donor liver reperfusion and in the postoperative period are also a major cause of cardiac events following LT. More data is needed to determine the predisposing factors for acute cardiac events, as well as the best screening and prevention methods in patients undergoing liver transplantation.

The primary aim of this study was to review our experience over a ten year period to determine the importance of acute cardiac events as a cause of mortality compared to noncardiac events at surgery and up to four months after LT using current cardiac preoperative screening strategies. Our hypothesis was that in the setting of eliminating nearly all patients with known CAD, and using myocardial stress testing to rule out ischemia in the rest, cardiac events and mortality would be uncommon compared to noncardiac mortality. A secondary aim was to see if asymptomatic patients <50 years old did not need cardiac screening as suggested by the AASLD.

## 2. Materials and Methods

We retrospectively reviewed the charts of all 393 patients who underwent LT at our institution between October 1st, 1999 and February 1st, 2008. Clinical data up to the four months postsurgery was available in all individuals. Patients were grouped based on age (<50 or ≥50 years old), the number of AASLD criterion (0 to 4) for cardiac risk (>50 years old, diabetes mellitus, current or past history of smoking, or family history of heart disease), prior history of CAD, and whether or not they underwent a preoperative cardiac stress test. The type of cardiac stress test performed was noted as a dobutamine stress echocardiogram (DSE), chemical nuclear perfusion study, or coronary angiogram. Approval for this study was obtained by the Mayo Clinic Arizona Institutional Review Board.

The definition of “high-risk” patients followed the AASLD guidelines [2] which included patients ≥50 years old, those with diabetes, past or present smoking, or with a family history of heart disease. The medical record and liver transplant operative report were reviewed for adverse cardiac events. Adverse cardiac events were defined as new myocardial infarction (MI) confirmed by serum troponin T and creatine kinase assays, new onset heart failure with a reduced LV ejection fraction, or an arrhythmia that required urgent treatment because of hemodynamic instability. These arrhythmias included severe bradycardia, asystole, ventricular tachycardia, or ventricular fibrillation. Serious adverse cardiac events were also classified by their timing as occurring intraoperatively, postoperatively, or in the post-operative period up to four months after liver transplant. Noncardiac deaths were defined as deaths not attributable to a primary cardiac cause but instead to medical or surgical complications within 4 months of LT. Classification of death as cardiac versus noncardiac was adjudicated independently by two board-certified cardiologists. Other clinical variables assessed included patient demographics and Model for End Stage Liver Disease (MELD) score.

## 3. Statistical Analysis

Statistical analysis was performed using SAS 9.1.3 software (SAS institute Inc. Cary, NC). Data are summarized as mean ± SD or number and percentage. Chi square and Fisher exact tests were used to compare pairs of categorical variables. Absolute and relative risk was calculated for cardiovascular events in patient groups with 95% confidence intervals calculated by the exact binomial method.

## 4. Results

**Figure 1:**
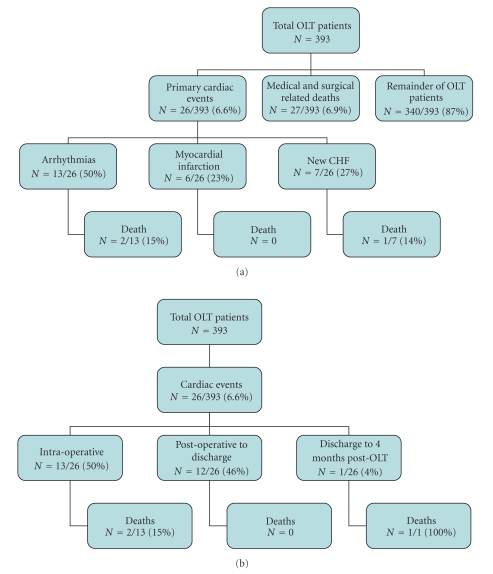
(a) All mortality and type of acute cardiac events after liver transplantation. CHF: congestive heart failure and LT: liver transplant. (b) Timing of acute cardiac events and when mortality occurred. LT: liver transplant.

**Figure 2:**
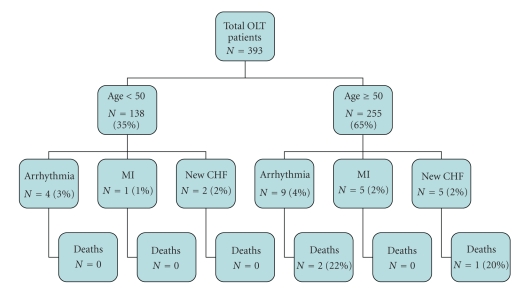
Acute cardiac events and mortality by age <50 or ≥50 years old in the LT patient study group. CHF: congestive heart failure, MI: myocardial infarction, and LT: liver transplant.

**Figure 3:**
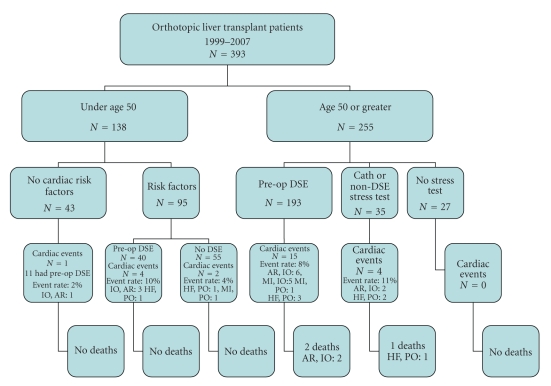
Acute cardiac events and mortality by age in the LT patient study group by age, cardiac risk factors, and results of preoperative cardiac stress test. AR: arrhythmia, cath: cardiac catheterization, DSE: Dobutamine stress echocardiogram, HF: heart failure, IO: intraoperative, MI: myocardial infarction, PO: perioperative period up until hospital discharge, and PD: time period from hospital discharge to 4 months posttransplant.

A total of 393 subjects underwent LT and were included in the analysis. Table 1 summarizes their clinical characteristics and Table 2 shows their risk factors by age groups. Figure 1 depicts the overall mortality, the number and type of adverse cardiac events, when the events occurred, and their outcome. Figure 2 shows the number and type of cardiac events and mortality in the LT patients by their age group of ≤ or > 50 years old. Figure 3 shows the cardiac events by age and risk factors and whether they had a preoperative cardiac stress test or angiogram.

In patients undergoing LT there were 30 deaths within the first four months after surgery for an overall mortality rate of 7.6%. Ninety percent of deaths (27/30) were due to surgical or medical complications. Figure 1(a) shows that there were 26 adverse cardiac events (6.6%). Three of the 30 total deaths were cardiac in nature (10%); 2 arrhythmias at the time of surgery and 1 new heart failure that occurred after hospital discharge. The overall cardiac mortality rate was very low at 0.8%.

Figure 1(b) shows the timing of the 26 adverse cardiac events. Twenty five of 26 events (96%) occurred before hospital discharge. Thirteen of these events (50%) occurred at the time of surgery. Twelve of these 13 events were cardiac arrhythmias (92%) and two patients could not be resuscitated and expired. There were 7 cases of new onset heart failure and 6 small non-*Q* wave myocardial infarctions by cardiac biomarker rise. The single patient who had an adverse cardiac event after hospital discharge developed new, severe heart failure with a reduced LV ejection fraction and died. Patients <50 years old had a lower rate of cardiac events than those over age 50 (5.1% versus 7.5%, *P* = .03), with only one myocardial infarction and no deaths.

The average MELD score in the patients with cardiac events was 23 ± 8. This was not different from patients who did not have cardiac events (*P* = .10) regardless of type. In the patients >50 years old who are by AASLD guidelines a high-risk group, 228 of 255 (89%) underwent and passed a cardiac screening test for CAD. In patients ≤50 who were considered high risk 40 of 95 (41%) underwent and had negative screening dobutamine stress echo (DSE). Eleven of 43 low-risk patients ≤50 years old (26%) also had negative DSE. There was no detectable difference in the incidence of cardiac events between high-risk patients who had a preoperative DSE (19 events (7.5% event rate) (*N* = 254)) compared with patients who did not undergo DSE testing (7 events (4.7% event rate) (*N* = 149) (*P* = .27)). The relative risk of four month cardiac events for high-risk patients by AASLD criteria compared with patients aged less than 50 with no cardiac risk factors was 3.071 (confidence interval 0.568–17.827) (Fisher exact test *P* = .34) (Figure 3). Table 3 shows the characteristics of patients who had a cardiac event. The median and average number of risk factors in patients who had a cardiac event (median = 1, average = 1.22 ± 0.80) did not differ from those who did not (median = 1, average = 1.03 ± 0.82) (*P* = .06).

## 5. Discussion

Acute cardiac events are recognized as an important cause of death in patients undergoing liver transplantation [11, 12], especially in those known to have preexistent CAD. In response, the AASLD has recommended preoperative cardiac stress testing to detect occult CAD in “high-risk” ESLD patients [2]. However, the scientific basis for this recommendation remains controversial [3–6] and many acute cardiac events occur in patients with few CAD risk factors and without obvious clinical, ECG, or serologic evidence of myocardial ischemia or infarction [10].

To reexamine the relation between possible occult CAD and acute cardiac events post-LT we reviewed the first 393 patients who underwent LT at our institution over a 10-year period. Because preexistent CAD is associated with increased cardiac mortality [11] only 11/393 patients (3%) that underwent liver transplantation had known preexistent CAD. Of the 350 LT patients who otherwise met “high-risk” AASLD criteria, 268 or 77% underwent a cardiac stress or imaging study to rule out significant coronary artery stenosis. All patients had follow-up till four months after their transplantation, after which many left our area and returned home.

The principal finding in our nearly 400 liver transplant recipients over a ten-year period is that by eliminating patients with preexistent CAD, and screening the majority of others with DSE, deaths due to acute, primary cardiac events at the time of surgery and up to four months post-LT are very uncommon (0.8%) compared to deaths related to medical and surgical complications (6.9%). Likewise, the overall cardiac event rate of 7% was quite low. The 138 patients <50 years old had an adverse event rate that was lower than the ≥ 50 age group (5.1% versus 7.5%), and regardless of AASLD cardiac risk factor score no fatal events occurred. This suggests that the current AASLD recommendation that “low-risk” patients <50 years of age could be expanded to all individuals in this younger patient group.

Cardiac arrhythmias that caused hemodynamic instability were included in our acute cardiac events even though they can be precipitated by metabolic and other factors independent of myocardial ischemia. A significant secondary finding in our study was that half of the 26 serious adverse cardiac events observed were life threatening arrhythmias, with 12 of 13 occurring in the operating room. These arrhythmias usually occurred at the time of, or after, donor liver reperfusion and resulted in two of the three cardiac fatalities that occurred. These events were also seen in the patients <50 years old, and were not predicted by the results of preoperative DSE cardiac stress testing. Preoperative DSE also did not predict the myocardial infarctions and new heart failure that were also seen in fewer numbers. The myocardial infarctions occurred immediately after surgery and during the hospitalized period but uniformly appeared to be small (by cardiac biomarkers and ECG findings) suggesting they may have been due to “demand ischemia” rather than a ruptured atherosclerotic plaque. All had a favorable prognosis.

Although the definition of an acute cardiac event varies in reports on the LT population, our overall incidence of short-term acute cardiac events of 7% with a mortality of <1% is lower than most other single-center experiences. For instance, in 117 patients undergoing LT a high cardiac event rate of 20% (including intraoperative hemodynamic events and postoperative troponin elevation) has been reported with no association between cardiac events and conventional preoperative risk factors, including age [4]. In a large group of LT patients studied in England between 1982 and 1998 (*n* = 1312), the three month incidence of cardiac events (MI, angioplasty, coronary artery bypass grafting, or cardiac arrest) was reported as 4.5% [13]. Recently, the incidence of new onset systolic heart failure following LT in patients without conventional risk factors was reported as 7% [14]. In a report from China, LT patients transplanted between 1993 to 2001 had a 7% incidence of MI and a 10% incidence of heart failure [15]. The most likely explanation for the low short-term cardiac event rate in the present study, and in particular MI, may have to do with patient selection and an aggressive DSE protocol. Nearly all patients with known CAD were turned down as candidates and during DSE dobutamine, fluids and sometimes atropine was used to reach a peak of >85% of maximal predicted heart rate or a peak heart rate times systolic blood pressure of >16,000 [10].

The most important risk factor for having a cardiac event with LT was being >50 years old. All three fatalities were seen in this group. Additional AASLD cardiac risk factors besides age did not increase the risk of cardiac events in our patients. Preoperative DSE versus no stress or imaging study also did not appear to relate to the incidence of cardiac events (Figure 3). However, without randomizing patients to have or forego standardized cardiac stress testing it is difficult to know how important preoperative screening for occult CAD is in the LT population. It may be that eliminating patients with known CAD who have presumably a large atheromatous plaque burden is more effective in reducing cardiac events than screening asymptomatic patients.

Intraoperative cardiovascular arrhythmias during LT surgery such as asystole, ventricular tachycardia, and ventricular fibrillation are well described, especially at the time of or after donor liver reperfusion [5, 16–21]. Although the causes of these events are unclear, myocardial ischemia may not necessarily be the most common trigger. Metabolic abnormalities, postreperfusion syndrome [16], pulmonary artery catheter induced arrhythmias [5], a prolonged ECG QT interval [17], or air embolus to the right heart associated with liver manipulation [18–21] have all been implicated. In the current study, where the incidence of “hard” ischemic events such as MI is low, these intraoperative arrhythmias stand out as being the most prominent cardiac events.

Potential substrate for serious cardiac arrhythmias in the ESLD population are common [9, 17]. A prolonged QTc on ECG in alcoholic patients with chronic liver disease is associated with a higher incidence of sudden death [7, 22]. ECG QTc prolongation of >440 ms has been reported in 47% of patients with cirrhosis compared to only 5% of control patients [23]. OTC prolongation and severity of ESLD (Child-Pugh score) has been established [24], and also related to markers of ventricular dysfunction including plasma BNP [25]. Chronotropic incompetence, or the inability to increase heart rate normally, is also associated with increased mortality in non-ESLD patients [22]. We have reported that chronotropic incompetence during DSE has the strongest relation to increased risk for cardiac events during, and in the 4 month period following LT [10].

Cardiac arrhythmias may also occur because of electrolyte disturbances during liver transplantation. Hypomagnesemia is almost universal during LT surgery [26] and hypokalemia and hypocalcemia are common [27].

Hemodynamic instability at the time of donor reperfusion is also well recognized and can lead to cardiac arrest [16, 28]. This postreperfusion syndrome is defined as a decrease in heart rate and mean arterial pressure of at least 30% for at least 60 seconds within the first five minutes after liver reperfusion [28]. Postreperfusion syndrome is thought to be caused by decreased systemic vascular resistance, myocardial depression, and blood loss [29, 30]. It is possible that this cardiac stress might contribute to the small myocardial biomarker release seen in LT patients which are often termed “demand” ischemia infarcts.

## 6. Limitations

Data in this study is from a single institution over nearly 10 years and has been collected in a retrospective manner which has well-recognized limitations compared to a prospective study. The results reflect elimination of nearly all candidates with known CAD which may account for our low MI and CHF rates. Our follow-up was limited to the four month period after liver transplantation because this represented the most complete data set. Most other studies regarding cardiac events following LT have measured long-term survivor risk. Although our single center data provides a more uniform approach to the LT population than a multicenter study, some groups analyzed were relatively small increasing the risk of sampling error. In many cases preoperative DSE did not reach target endpoints and, therefore, could be labeled as an inadequate stress [10]. For these reasons our results do not help resolve the controversy of the need for, or best way to perform a cardiac stress test in the older LT candidates.

The low overall incidence of CV events and death with a most events being intraoperative and postoperative cardiac arrhythmias in our population was the underappreciated major finding in this study. This could be because many studies focus on longer term follow-up. Detailed information about the donor liver and intraoperative variables such as ECG abnormalities, hemodynamics (especially hypotension post reperfusion), and medications used would be important to better understand the causes of these serious intraoperative arrhythmias.

## 7. Conclusion

By using the current screening strategy supported by the AASLD, deaths due to acute, primary cardiac events at the time of surgery and up to four months post-LT are very uncommon when compared to deaths related to medical and surgical complications. In patients over 50, intraoperative arrhythmias are major causes of acute cardiac events. As preoperative DSE cardiac stress testing did not predict these events, further study on the factors which cause myocardial electrical instability at the time of liver transplantation appear warranted.

## Figures and Tables

**Table 1:** Liver transplant patient characteristics.

*N* = patients	393
Age (years)	52.0 ± 10.1 (range 17–72)
Male	267 (68%)
Diabetes Mellitus	94 (24%)
Hypertension	85 (21%)
History of smoking	240 (58%)
Smoking within 1 year of transplant	89 (23%)
Known Coronary Artery Disease	11 (3%)
MELD score at time of transplant	21 ± 8
Cold Ischemic Time (hours)	5.9 ± 2.5
Etiology of Liver Failure	
Hepatitis C	161 (41%)
Alcohol-related cirrhosis	51 (13%)
Hepatocellular carcinoma	50 (12%)
Cryptogenic idiopathic cirrhosis	24 (6%)
Primary sclerosing cholangitis	20 (5%)
Primary biliary cirrhosis	12 (3%)
Other	65 (17%)

MELD: model for end stage liver disease.

**Table 2:** Cardiac events in the liver transplant population at MCA (*N* = 393).

	*N*	Events	Event Rate	Deaths	Event Type and Period					
					No Death			Death		
					IO	PO	PD	IO	PO	PD
*Age *< 50	138	7	5%	0	4	3	0	0	0	0
No Cardiac Risk Factors	43	1	2%	0	AR: 1	0	0	0	0	0
Cardiac Risk Factors	95	6	6%	0	3	3	0	0	0	0
DSE	40	4	10%	0	AR: 3	HF: 1				
No DSE	55	2	4%	0		MI: 1, HF: 1				

*Age* > 50	255	19	8%	3	7	9	0	2	0	1
DSE	193	15	8%	2	AR: 4; MI: 1	MI: 4; HF: 3; AR: 1	0	AR: 2	0	
Non-DSE stress/cath	35	4	11%	1	AR: 2	HF: 1				HF: 1
No stress test	27	0	0%	0						

AR: arrhythmia, cath: cardiac catheterization, DSE: Preoperative dobutamine stress echocardiogram, HF: heart failure, IO: intraoperative, MI: myocardial infarction, PO: perioperative period up until hospital discharge, and PD: time period from hospital discharge to 4 months posttransplant.

**Table 3:** Cardiac events: Patient characteristics.

*n*	26
Age (years)	55 ± 8
Male	19 (73%)
Diabetes mellitus	6 (23%)
Hypertension	11 (42%)
History of smoking	15 (58%)
Known Coronary Artery Disease	1 (4%)
MELD score at time of transplant	23 ± 8
Cold Ischemic Time (hours)	6.0 ± 2.0

MELD: model for end stage liver disease.

## Abbreviations

AASLD: The American Association for the Study of Liver Diseases
CAD: Coronary Artery Disease
DSE: Dobutamine Stress Echocardiography
ESLD: End Stage Liver Disease
FRS: Framingham Risk Score
LT: Liver Transplantation
MELD: Model for End Stage Liver Disease
MI: Myocardial Infarction.
